# Fibulin-2 Facilitates Malignant Progression of Hepatocellular Carcinoma

**DOI:** 10.5152/tjg.2023.22134

**Published:** 2023-06-01

**Authors:** Xinyan Hu, Tianze Liu, Luting Li, Hairun Gan, Tiancheng Wang, Pengfei Pang, Junjie Mao

**Affiliations:** 1 Department of Interventional Medicine, The Fifth Affiliated Hospital, Sun Yat-sen University, Zhuhai, China; 2 Guangdong Provincial Key Laboratory of Biomedical Imaging, The Fifth Affiliated Hospital, Sun Yat-sen University, Zhuhai, China; 3 Guangdong Provincial Engineering Research Center of Molecular Imaging, The Fifth Affiliated Hospital, Sun Yat-sen University, Zhuhai, China; 4 Institute of Interventional Radiology, Sun Yat-sen University, Zhuhai, China; 5 The Cancer Center, The Fifth Affiliated Hospital, Sun Yat-sen University, Zhuhai, Guangdong Province, China

**Keywords:** Fibulin-2, hepatocellular carcinoma, oncogene, MAPK pathway

## Abstract

**Background:**

Identification of biomarkers to assist in the clinical management of hepatocellular carcinoma represents an urgent requirement. Fibulin-2 is known to contribute to the development and progression of various cancer types. This research investigated the role of fibulin-2 in hepatocellular carcinoma and explored the possible mechanisms.

**Methods:**

The expression of fibulin-2 in hepatocellular carcinoma was measured by bioinformatic analysis and confirmed by western blot and immunohistochemical staining in cell lines or patients’ samples. The clinicopathologic features of hepatocellular carcinoma patients was analyzed. Cell viability assays were used to explore the role of fibulin-2 on proliferation in hepatocellular carcinoma. Western blot was conducted to uncover changes of protein expression of Ras-MEK-ERK1/2 pathway when Fibulin-2 was overexpressed or silenced. Flow cytometry analyses were used to determine the roles of fibulin-2 in the function of apoptosis and cell cycle. Subcutaneous xenograft mouse models showed the tumor growth pattern after fibulin-2 silence in vivo.

**Results:**

We reported the upregulation of fibulin-2 in most hepatocellular carcinoma tissues and cells lines. Fibulin-2 promoted the proliferation of hepatocellular carcinoma cells in vitro by regulating Ras-MEK-ERK1/2 signaling pathway, whereas knockdown of fibulin-2 incurred the opposite effect on proliferation. Consistently, knockdown of fibulin-2 resulted in increased apoptosis and induced growth arrest during the G0/G1 phase transition. In vivo xenograft assessment confirmed that knockdown of fibulin-2 inhibited hepatocellular carcinoma tumor growth.

**Conclusions:**

Fibulin-2 exhibited tumor promotor activities in malignant progression of hepatocellular carcinoma. The results of the study highlighted the potential of fibulin-2 to be utilized as a promising biomarker and therapeutic target for hepatocellular carcinoma.

**Main Points**

- Fibulin-2 is mostly upregulation in hepatocellular carcinoma (HCC) cell lines and tissues.
- Fibulin-2 facilitates proliferation and influences the Ras-MEK-ERK1/2 pathways in HCC.
- Knockdown of Fibulin-2 induces cell apoptosis and growth arrest during the G0/G1 phase.

## Introduction

As one of the most frequently diagnosed and deadly cancer types, hepatocellular carcinoma (HCC) accounts for 4.5% of all new cancer cases and more than 8.3% of cancer-related deaths in 2020.^1^ In spite of the substantial advances in treatment including surgery, interventional therapy, radiotherapy, etc., the prognosis of HCC remains far from satisfactory in a real-world scenario due to complex pathogenic mechanisms and high heterogeneity. The 5-year survival rate for HCC is merely estimated to be 18% in advanced-stage patients despite the use of systemic treatment strategies that involve immune checkpoint inhibitors and targeted therapy.^2^ Of all the etiological factors of HCC, cirrhosis due to chronic hepatitis B virus infection is particularly prominent in Asian populations, and multiple factors associated with cirrhosis also activate proliferation of tumors.^3^ The ambiguous molecular mechanisms of the evolution from cirrhosis to HCC have prompted a deeper mechanistic exploration of the tumorigenesis and progression of HCC at the molecular level, thus providing more inspiration for the pursuit of new biomarkers and potential therapeutic targets.

Featured by progressively extracellular matrix (ECM) remodeling and stiffened, cirrhosis leads to altered substrate stiffness which activates epithelial mesenchymal transition (EMT) and enhances cell migration.^4^ The members of the fibulin family usually encode ECM proteins that bound together to form a complex network, which is known to act as substrates and physical scaffolds associated with cancer adhesion, migration, and invasion.^5^ Assessment of these family members provided evidence for their essential roles in the occurrence and progression of multiple tumor types. For example, fibulin-1 and fibulin-5 are known to be associated with tumorigenesis and metastasis in HCC.^6,7^ Located between the elastin core and the microfibrils, FBLN2 acts as a calcium-binding glycoprotein binding and interacting with a variety of ECM components.^8,9^ Previous studies highlighted its involvement in the elastogenesis of blood vessels, which is most seen in diseases like atherosclerosis, acute aortic dissection, and hypertension that are closely related to ECM reorganized and desmoplasia.^10,11^

In recent times, several studies provided accumulating evidence for tumor-related properties of FBLN2 in various tumor types. Physically, the binding of FBLN2 to cell adhesion molecules enhances the stability of ECM, and the absence of FBLN2 is associated with disruption of basement membrane integrity and early invasion of tumor cells.^12^ Furthermore, FBLN2 regulates ECM composition by modulating ECM-mediated extracellular–intracellular signaling, which further affects the stability and integrity of the basement membrane and thus participates in carcinogenesis.^12^ It is reported that FBLN2 plays an oncogenic role in urothelial carcinoma, pancreatic cancer, and lung adenocarcinoma.^8,9,13^ In breast cancer, Kaposi’s sarcoma, and nasopharyngeal carcinoma, it was shown to exhibit antitumor activities.^14-16^ Moreover, emerging evidences suggested that FBLN2 acts as a key pro-fibrotic factor. In fibrotic and cirrhotic livers, FBLN2 acts as a reliable cellular marker of myofibroblasts around the portal vein.^17^ Besides this, an increased expression of FBLN2 was reported in experimental rat liver cirrhosis, and greater induction of FBLN2 was detected in human liver cirrhosis.^18^ However, some researches hypothesized that FBLN2 promotes the development of HCC rely on database analyses.^19-21^ Crucial experimental evidence to verify the function of FBLN2 in HCC is still scarce and there is limited knowledge about the regulatory role of FBNL2 in the progression of HCC.

Given the role of FBLN2 in cirrhosis and other tumors and the paucity of research in HCC, our study aimed to evaluate the expression of FBNL2 in HCC. We proposed to further investigate the role of FBLN2 in the progression of HCC and preliminarily explore its possible mechanisms. The findings of the study revealed the potential clinical significance of FBNL2 as a biomarker and therapeutic target in HCC, which may provide new ideas for targeted therapy of HCC in the coming years.

## Materials and Methods

### Cell Lines

HepG2 (HB-8065™), PLC/PRF/5 (CRL-8024™), SNU398 (CRL-2233™), and SNU449 (CRL-2234™) were obtained from the American Type Culture Collection (ATCC, Manassas, Va, USA). L02, Bel-7402, and HCCLM3 were purchased from the Shanghai Cell Bank of the Chinese Academy of Sciences (Shanghai, China). All cells were grown in Dulbecco’s modified Eagle medium (GIBCO, Canada, C11995500BT) supplemented with 10% fetal bovine serum (FBS) and 100 U/ml penicillin/100 µg/ml streptomycin solution (Thermo Fisher) in a humidified atmosphere containing 5% CO_2_ at 37°C.

### Clinical Samples and Immunohistochemical Staining

Samples of HCC and adjacent liver tissue with normal histology were collected during surgery at the Fifth Affiliated Hospital of Sun Yat-sen University. This study was approved by the ethics committee of the Fifth Affiliated Hospital of Sun Yat-sen University ([2019] No. L127-1), and all patients have signed informed consents. The privacy rights of human subjects had always been observed. A total of 9 tissues of HCC patients were embedded in low-melting point paraffin and cut into 4-μm thick tissue slides. All tissue slides were deparaffinized, rehydrated, and boiled in citrate buffer (10 mM, pH 6.0) under high pressure for 10 minutes for antigen retrieval. Each section was incubated with a peroxidase inhibitor, and then blocked with goat serum. Anti-FBLN2 (Sigma, USA, SAB2702003) was diluted 1:200 and incubated in a humidified container at 4°C overnight. After incubated with an HRP-conjugated secondary antibody at 25°C for 30 minutes, diaminobenzidine kit (ZSGB-BIO, China, ZLI-9017) was used for chromogenic reaction. Then, all images were visualized and captured by Pannoramic 250 Flash II digital scanner (3DHISTECH Ltd., Budapest, Hungary) and H-score was automatically calculated for all tissues using Case Viewer (3DHISTECH Ltd., Budapest, Hungary) as: *H*-score = staining intensity score × percentage score.

### Cell Transduction

The full-length cDNA of the FBLN2 (pCDH-CMV-Homo-FBLN2-EF1-puro, Guangzhou IGE Biotechnology LTD, China) was cloned into lentiviral expression vectors. Fibulin-2 stable knockdown cell lines were transduced with lentiviral vectors (FBLN2-Homo-1839 and FBLN2-Homo-2683 for shRNA, target sequences: 5′-GCTGCACCACGGAGAGTTTCA-3′ and 5′-GCAACTGTGTGGACATCAACG-3′, respectively) purchased from GenePharma (Suzhou, China). For transduction, according to the manufacturer’s instructions, cells were transfected with FBLN2 lentivirus (MOI = 10) in serum-free medium with the presence of 10 μg/mL polybrene for 24 hours. Then, cells were rinsed and replaced with fresh medium with 10% FBS and 1% penicillin/streptomycin solution. Transduced cells were then selected with puromycin (1 μg/mL) for 7-14 days and maintained with puromycin-medium for routinely selection.

### Western Blot

The cells or tissues were lysed with ice-cold RIPA buffer (Sigma) containing protease inhibitor cocktail (Roche) for 30 minutes and subsequently centrifuged at 14 000 rpm at 4°C for 20 minutes. The supernatant containing protein were isolated, and the protein concentration was detected using a BCA kit (Beyotime Biotechnology, Shanghai, China, P0010) followed by boiling with SDS loading buffer for 10 minutes at 100°C. After the electrophoresis and transferation of the protein samples according to standard procedures, PVDF membranes containing the proteins were blocked using 5% skim milk for 1 hour and incubated with primary antibodies overnight. The primary antibodies used in this study were as follows: anti-FBLN2 (Sigma, USA, SAB2702003), anti-β-actin (Cell Signaling Technology, USA, 4970S), anti-p-ERK1/2 (Cell Signaling Technology, USA, 4370), anti-ERK1/2 (Cell Signaling Technology, USA, 4695), anti-p-MEK (Cell Signaling Technology, USA, 3958), anti-MEK (Cell Signaling Technology, USA, 9126), anti-RAS (Cell Signaling Technology, USA, 67648T), and anti-GAPDH (Cell Signaling Technology, USA, 5174S). After washed with washing buffer (tris buffered saline solution with tween 20) for 3 times, membranes were incubated with secondary antibodies (1:1000, EARTHOX, E030110-01 or E030120-01). The chemiluminescence reaction was performed using the Enhanced ECL Kit (Gension, Guangzhou, China).

### Real-Time Quantitative Polymerase Chain Reaction

According to the manufacturer’s instruction, total RNA was extracted from the cells using Total RNA Kit I (Omega Biotek, China, Cat#R6834-02). A total of 1000 ng of total RNA was applied to perform cDNA synthesis using All-in-OneTM Qpcr Mix (GeneCopoeia^TM^, Md, USA). Real-time quantitative PCR (RT-qPCR) was performed using SYBR Green Mix (BioRad, Hercules, Calif, USA), and followed by detection using Bio-Rad CFX96 and analysis via Bio-Rad Manager software (BioRad, Hercules, USA). Each gene expression level was normalized to the expression of GAPDH. Primer sequences are as follows: FBLN2-F:5′-CTGCTACAAGGCACTCACCTGT-3′; FBLN2-R:5′- GTAGAAGGAGCCCTTGGTGTTC-3′ (Guangzhou IGE Biotechnology LTD, China); GAPDH (GeneCopoeia, Maryland, USA, Hs-QRP-20169).

### Cell Viability Assay

For cell viability assay, stable transfected HCC cells were plated in 96-well plates at 1000 cells per well. After 72 hours of culture in an incubator, medium was removed and replaced with 100 μL Cell Counting Kit-8 (KeyGEN BioTECH Corp., Jiangsu, China) for 2 hours. The absorbance at 490 nm was measured by multifunctional microplate reader (Safire, TECAN) and compared via GraphPad Prism software (V.7.0). Average percentages of cell viability were then calculated.

### Colony-Formation Assays

After cell transduction, stable HCC cells were grown in 6-well plates at 1200 cells per well and changed to 2 mL of fresh media with 10% FBS every 2 days. After 14 days, surviving colonies were fixed with methanol for 1 hour and visualized with 0.1% crystal violet solution. After washing with ddH_2_O, the colonies were photographed, and the number of colonies was counted via Image J Image Processing software.

### Flow Cytometry Analysis

Cell apoptosis analysis was performed using the Annexin V-APC/propidium iodide (PI) apoptosis detection kit (KGA1030-50, KeyGEN BioTECH, Nanjing, China). According to the manufacturer’s instructions, HCC cells were harvested, washed with PBS, resuspended in binding buffer, and then stained with Annexin V-APC and PI buffer for 30 minutes at room temperature in the dark. Then, the ratios of apoptotic cells were measured by flow cytometry (CytoFLEX LX, Beckman Coulter, CA).

Cell cycle analysis was done using the cell cycle kit (KGA512, KeyGEN BioTECH, Nanjing, China) following the manufacturer’s instructions. Hepatocellular carcinoma cells were collected and washed with PBS for 3 times. Then, cells were carefully resuspended and fixed with 70% ethanol overnight at 4°C. Cells were stained with PI/RNase buffer for 20 minutes at room temperature and shielded from light. Cell cycle of the stained HCC cells were then measured immediately by flow cytometry (CytoFLEX LX, Beckman Coulter, CA) and percentages of cells in different cell cycle phases were calculated.

### Animal Studies

Study was performed in accordance with the ARRIVE guidelines and approved by the Animal Ethics Committee of Sun Yat-sen University (Animal Ethics Committee approval #00163). Four-week-old BALB/c female nude mice (Beijing Vital River Laboratory Animal Technology Co., Ltd, China) were housed in standard cages with 12-hour light/dark cycles and ad libitum access to food and water. All nude mice were subcutaneously injected 2 × 10^6^ indicated stable SNU398 cells resuspended with 100 μL PBS. The tumor volume was measured by digital caliper every 3 days (Volume =  width^2^ × length × 0.52). Three weeks later, all mice were anesthetized under pentobarbital sodium and humanely sacrificed, tumors were harvested, and imaged. The weights of tumors were then measured and the average weights were calculated.

### Statistical Analysis

All statistical analyses were performed using GraphPad Prism software (V.7.0). Ratios of apoptotic cells and cell cycle rates of the stained HCC cells were analyzed using 2-tailed chi-square test. Additionally, numeric variables were presented as the mean ± SD. Assessment of the relative expression of mRNA and protein, number of colonies, absorbance of Cell Counting Kit-8 assay, immunohistochemistry (IHC) score, and weight and volume of tumors between 2 groups were carried out by 2-tailed Student’s *t*-test. Statistical significance was defined as *P*-values less than .05. *P < .05; **P < .01; ***P < .001; ****P < .0001.

## Results

### Fibulin-2 Is Highly Expressed in Hepatocellular Carcinoma

**Figure 1.**
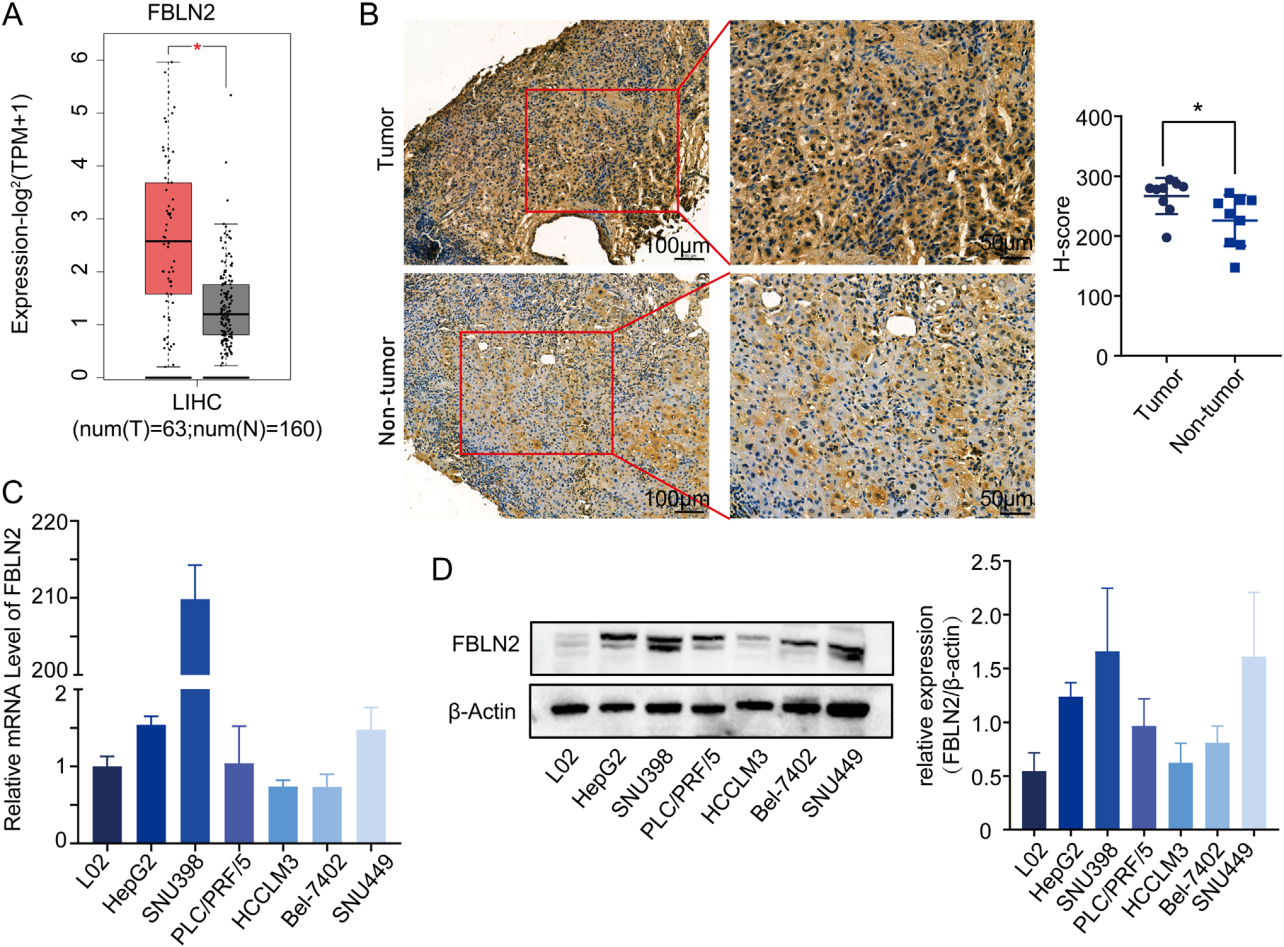
Fibulin-2 (FBLN2) is overexpressed in HCC tissues and cells. (A) A box plot in the GEPIA2 database demonstrating the FBLN2 expression in 63 HCC tumors (red plot) and 160 normal liver tissues (gray plot). (B) Representative images of IHC staining of FBLN2 were shown on the left and *H*-score statistics was shown on the right panel. (C) The mRNA levels of FBLN2 in different HCC cell lines and a normal hepatocyte cell line. (D) The expression levels of FBLN2 protein in 6 HCC cell lines and a normal human hepatocyte cell line. *P < .05.

To analyze the expression profile of FBLN2, the online database Gene Expression Profiling Interactive Analysis 2 (GEPIA2, http://gepia2.cancer-pku.cn/) was used. Data from the Cancer Genome Atlas and the genotype-tissue expression projects revealed that FBLN2 was upregulated in HCC as compared to normal liver samples (shown in Figure 1A). To further explore the expression of FBLN2 in HCC patient samples, IHC was conducted for 9 pairs of HCC tissues and adjacent non-cancerous tissues. As shown in Figure 1B, higher expression of FBLN2 was observed in HCC tumor tissues (*H*-score: 266.76 ± 28.41) than the liver tissues present adjacent to the tumors (*H*-score: 227.37 ± 41.69) (*P* < .05). According to analysis of clinical characteristics (Supplementary Tables 1 and 2), the high expression of FBLN2 was significantly correlated with liver cirrhosis history. The groups of high and low expression of FBLN2 differed in age which might be caused by the small number of samples (Supplementary Table 2). Following this, we analyzed the expression pattern of FBLN2 in different HCC cell lines. Fibulin-2 expression in human HCC cell lines (HepG2, PLC/PRF/5, SNU398, SNU449, Bel-7402, and HCCLM3) and normal human hepatocytes (L02) was detected by RT-qPCR. When compared to L02, FBLN2 was found to be upregulated in most HCC cell lines at mRNA (shown in Figure 1C). Western blot was conducted to explore the expression in different cell lines. The relative gray scale value (FBLN2/β-actin) indicated that the expression of FBLN2 was higher in all HCC cell lines than in normal hepatocytes (shown in Figure 1D). These results illustrated upregulated expression of FBLN2 in HCC tissues and cell lines, implying its possible association with HCC progression.

### Fibulin-2 Facilitates Proliferation of Hepatocellular Carcinoma Cells Through Increasing the Ras-MEK-ERK1/2 Signaling Cascade

**Figure 2.**
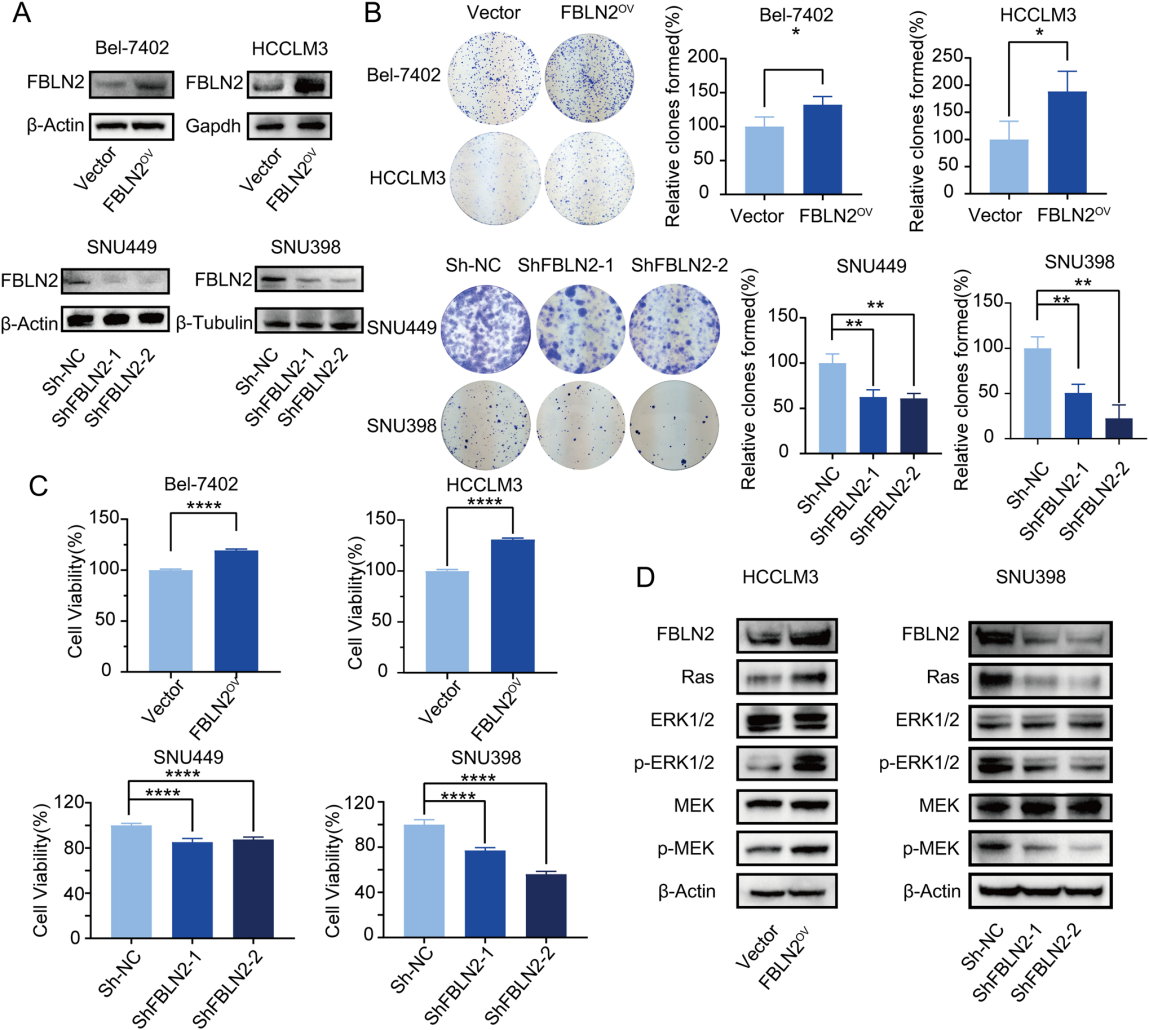
Fibulin-2 (FBLN2) induces HCC proliferation through increasing the Ras-MEK-ERK1/2 pathway. (A) The expression of FBLN2 protein in HCC cells after stable transduction. (B) The stably indicated HCC cells were subjected to colony-formation assay. Left panel: representative images of the colony formation; right panel: quantification for the number of clones. (C) Cell viability analysis shows that FBLN2 promoted HCC cell proliferation. (D) Western blotting shows the protein expression of the MAPK signaling pathway in HCC with the FBLN2 overexpression or downregulation. *P < .05; **P < .01; ****P < .0001.

**Supplementary Figure 1.**
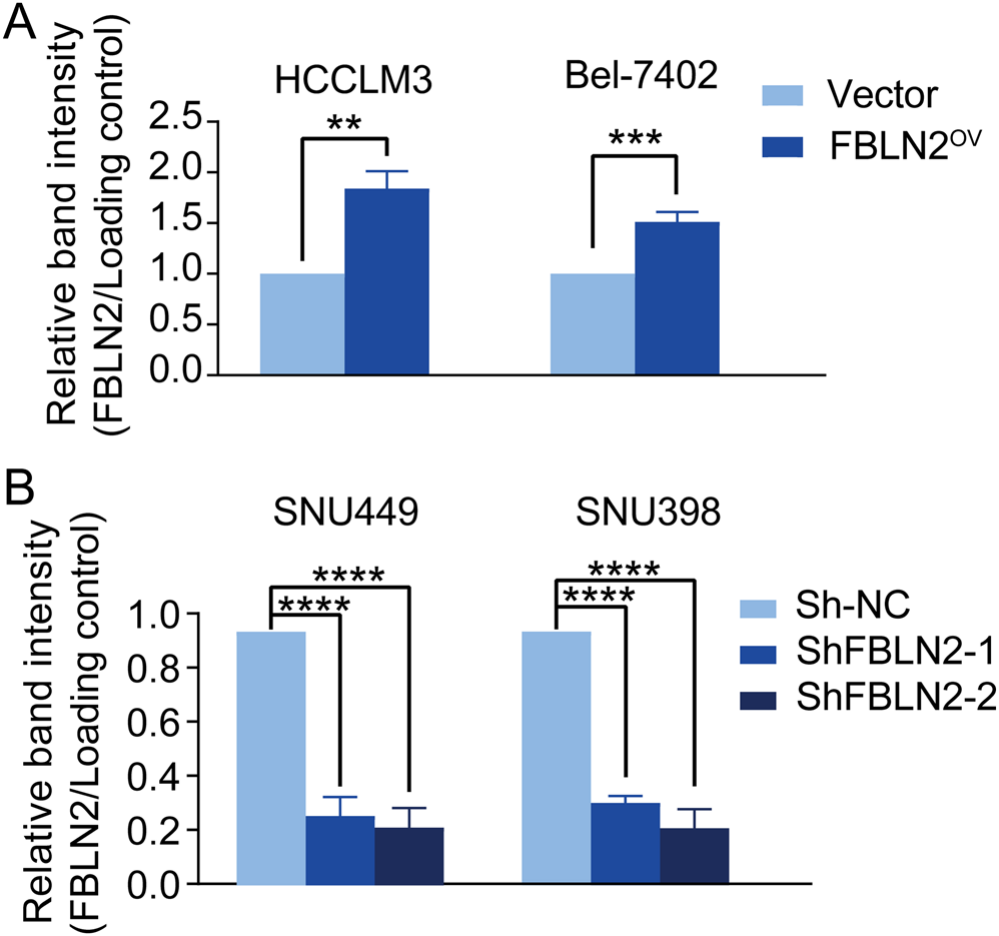
Supplementary Figure 1. FBLN2 protein was unregulated or downregulated in HCC cells after stable transduction. Graphs of densitometric quantification of FBLN2 in (A) HCCLM3, Bel-7402, (B) SNU449, or SNU398 cells after stable transduction. β-actin, GAPDH, and β-tubulin were used as loading control depending on circumstances. The results were shown as means ± SD. **P < .01; ***P < .001; ****P < .0001.

To further investigate the functional role of FBLN2 in HCC, the HCC cell lines Bel-7402, HCCLM3, SNU449, and SNU398 were transduced with lentiviral expression vectors and stable overexpression or knockdown of FBLN2 was confirmed at the protein level (shown in Figure 2A and Supplementary Figure 1). Fibulin-2 overexpression markedly fostered the colony-formation ability in Bel-7402 and HCCLM3 cells, while FBLN2 knockdown remarkably reduced colony formation in SNU449 and SNU398 cells (shown in Figure 2B). Likewise, overexpression of FBLN2 was found to be associated with increased proliferation of Bel-7402 and HCCLM3 cells, whereas stable downregulation of FBLN2 conspicuously compromised the cell viability in SNU449 and SNU398 cells (shown in Figure 2C).

**Supplementary Figure 2.**
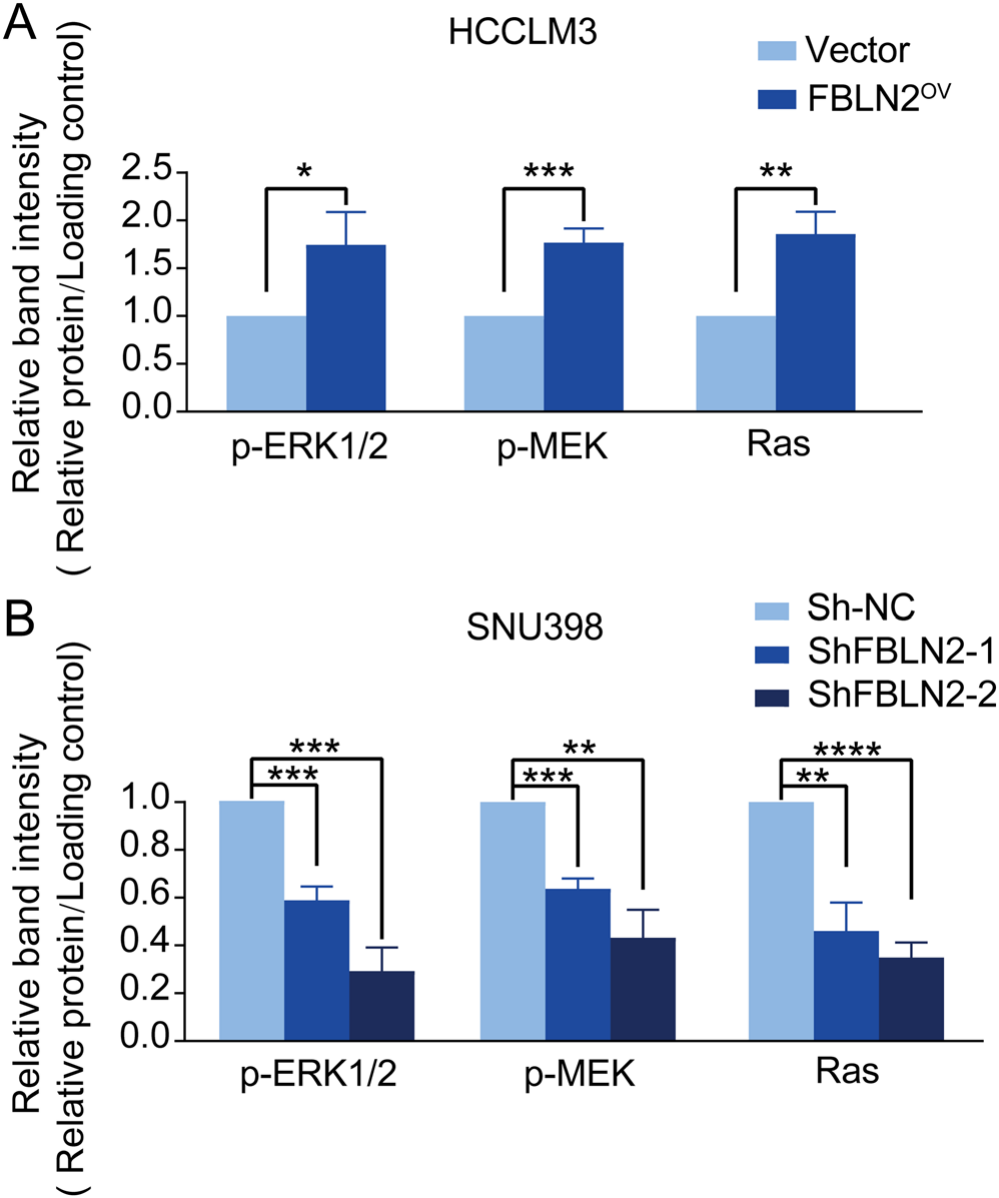
Supplementary Figure 2. The expression of Ras-MEK-ERK1/2 pathway in HCC was affected with the FBLN2 overexpression or downregulation. Graphs of densitometric quantification of the expression of p-ERK1/2, p-MEK and Ras in (A) HCCLM3 cells in the presence of FBLN2 overexpress or in (B) SNU398 cells that stably transduced with shRNAs (shFBLN2-1 or shFBLN2-2) or control shRNA (sh-NC). β-actin, GAPDH, and β-tubulin were used as loading control depending on circumstances. The results were shown as means ± SD. *P < .05; **P < .01; ***P < .001; ****P < .0001.

Considering the extensive crosstalk between Ras-MEK-ERK1/2 mitogen-activated protein kinase (MAPK) signaling cascade and cancer cell growth,^20^ we further investigated whether this pathway was responsible for FBLN2-mediated cell growth and proliferation in HCC. Interestingly, overexpression of FBLN2 promoted the activity of Ras and phosphorylation of ERK1/2 and MEK. In comparison to this, downregulation of FBLN2 resulted in the inhibition of the Ras-MEK-ERK1/2 signaling pathway (shown in Figure 2D and Supplementary Figure 2). Altogether, these results suggested that FBLN2 exerted a promoting effect to facilitate HCC cell proliferation, which was mediated via regulation of the Ras-MEK-ERK1/2 signaling cascade.

### Fibulin-2 Knockdown Induces Apoptosis and Cell Cycle Arrest in Hepatocellular Carcinoma Cells

**Figure 3.**
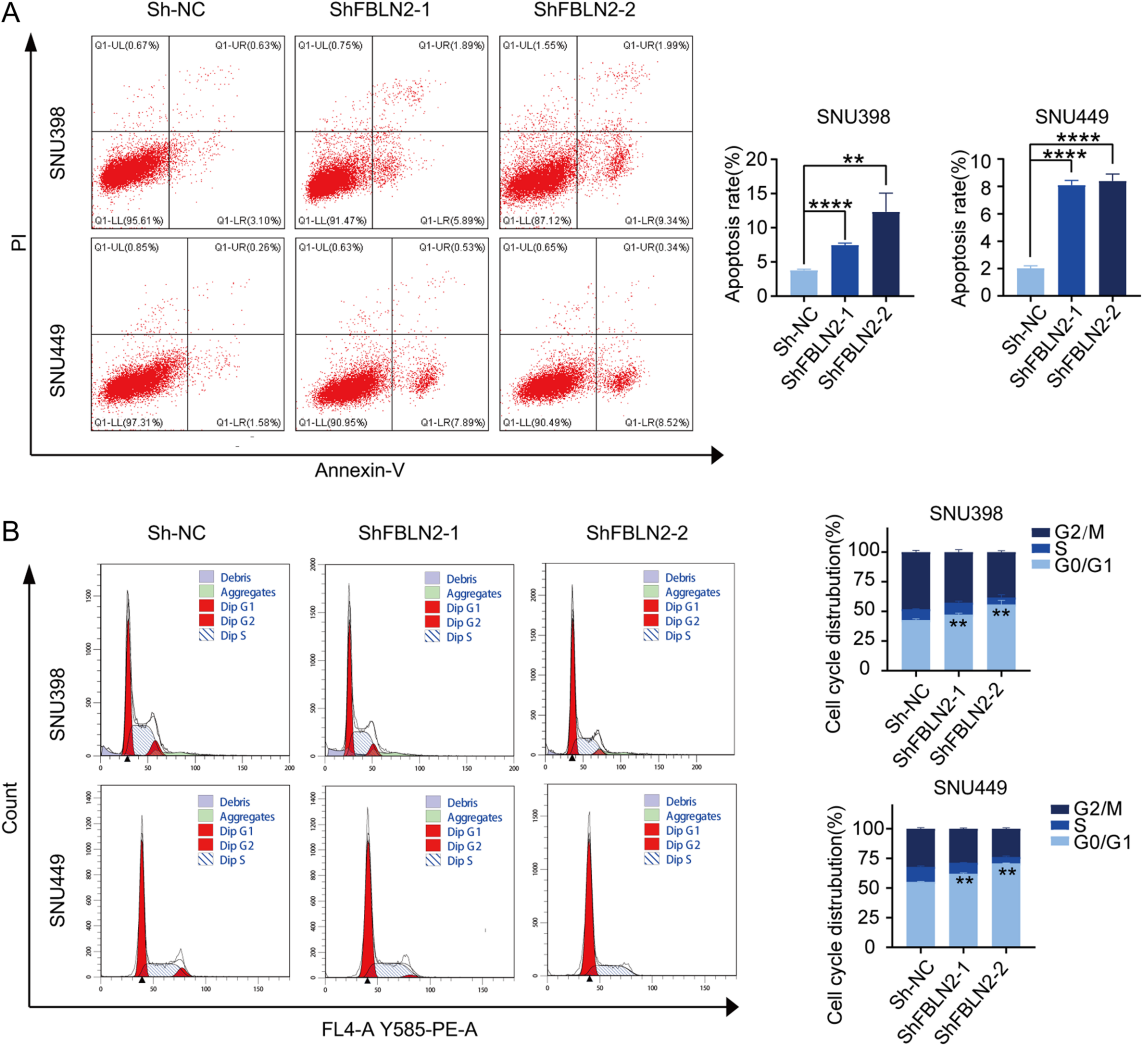
Fibulin-2 (FBLN2) suppresses apoptosis and induces cell cycle arrest in HCC cells. (A) Apoptosis was examined by flow cytometry. Left panel: gating strategy; right panel: quantification of the apoptosis rate. (B) Cell cycle distributions and the percentages of cells in the G0/G1, S, and G2/M phases in HCC cells with FBLN2 knockdown. Left panel: gating strategy; right panel: frequencies of cells in different phases of the cell cycle.

Flow cytometry was performed to assess the status of apoptosis and cell cycle in FBLN2-transduced HCC cells. As illustrated in Figure 3A, knockdown of FBLN2 resulted in obvious upregulation of apoptotic cells. Both in SNU449 and SNU398 cells, the apoptosis rates of FBLN2-knockdown cells were multiplied more than 2 times compared with the control group. In addition to this, knockdown of FBLN2 induced cell cycle arrest at the G0/G1 stage in HCC cells (shown in Figure 3B), which meant that FBLN2 knockdown induced growth arrest in HCC cells. These effects of FBLN2 on apoptosis and cell cycle further confirmed its tumor-promotive role in HCC cells.

### Fibulin-2 Knockdown Constrains Hepatocellular Carcinoma Tumor Growth In Vivo

**Figure 4.**
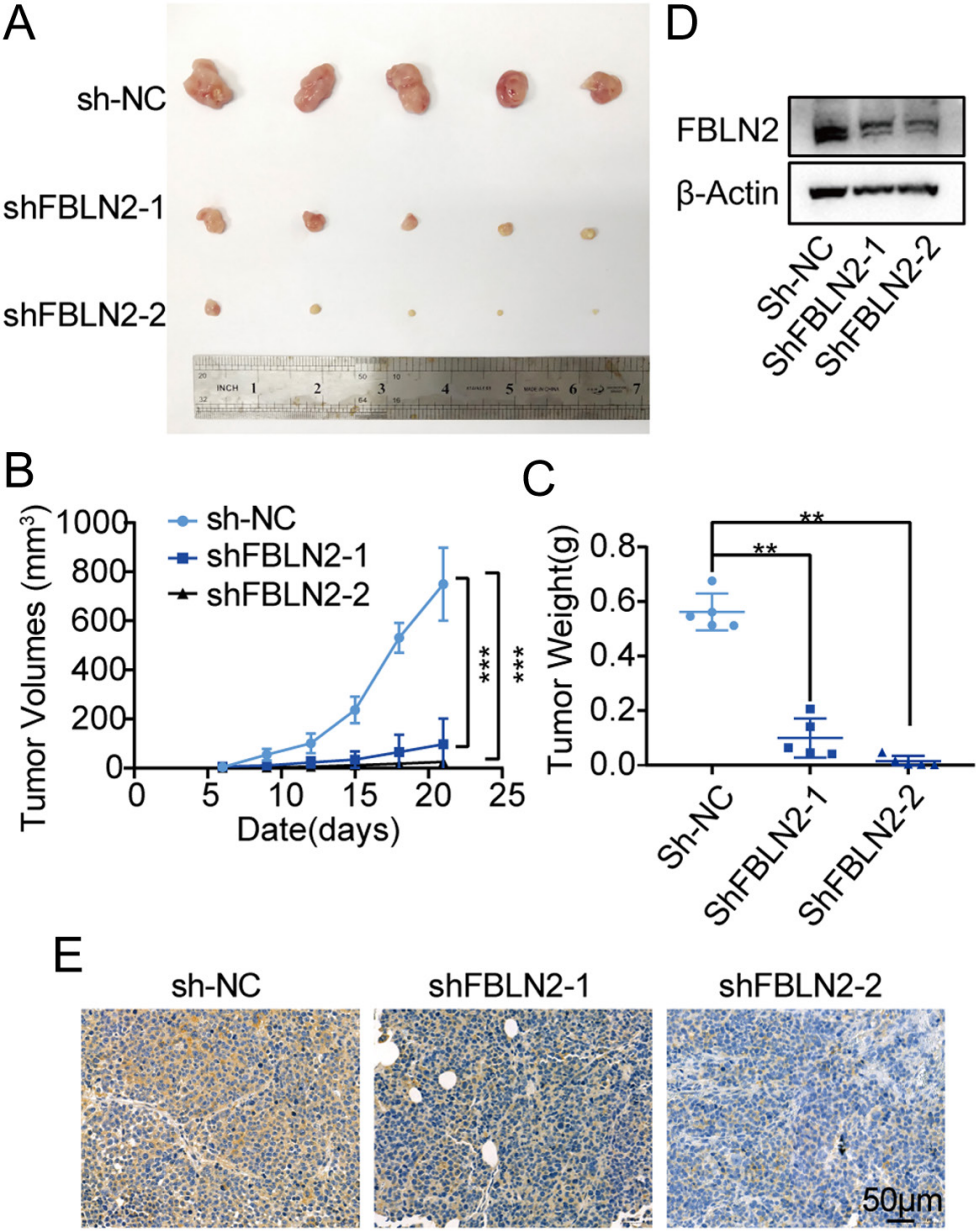
Silence of FBLN2 represses HCC growth in vivo. (A) Graphs of subcutaneous tumors derived from A549 cells that stably transduced with shRNAs (shFBLN2-1 or shFBLN2-2) or control shRNA (sh-NC). (B) The expression of FBLN2 protein extracted from subcutaneous tumors was analyzed by the western blot. (C) Representative images of the IHC staining of FBLN2 in subcutaneous tumors. The volume (D) and weight (E) of subcutaneous tumors with stably transduction were measured. FBLN2, fibulin-2. **P < .01; ***P < .001.

To further validate the functional effects of FBLN2 downregulation on HCC progression in vivo, subcutaneous tumor models were utilized. When compared with control, knockdown of FBLN2 was found to exhibit significant antitumor abilities (shown in Figure 4A). Experiments in vivo showed that FBLN2 knockdown in the subcutaneous tumor resulted in an obvious reduction in tumor volume (shown in Figure 4B). In addition to this, the tumor weight was recorded to be notably lower in the knockdown group as compared to the control group (shown in Figure 4C). IHC of tissues and proteins extracted from subcutaneous tumors were further used to confirm stable downregulation of FBLN2 (shown in Figure 4D and 4E). In concordance with the results of in vitro experiments, in vivo assessment indicated that downregulation of FBLN2 inhibited tumor growth in the mice model, which further verified the oncogenic role of FBLN2 in HCC.

## Discussion

Primary HCC is highly heterogeneous, with markedly different molecular features in different patients and even within the same HCC tissue. Current clinical and pathological staging cannot accurately assess the heterogeneity of HCC, the combined assessment based on morphological and molecular features better reflects the biological characteristics and prognosis of HCC. Progress in molecular biology has dramatically advanced our knowledge of the molecular mechanisms of oncogenesis and progression, which in turn has provided new insights for the development of novel targeted drugs as potential HCC treatments. Encouragingly, a variety of targeted therapies, including vascular endothelial growth factor (VEGF),^22^ hepatocyte growth factor,^23^ and fibroblast growth factor receptor 4 (FGFR4),^24^ have been approved as promising treatments for advanced HCC.

Indeed, cirrhotic or non-cirrhotic hepatocytes dysfunction related to chronic hepatitis B virus infection has been previously shown to act as the leading cause of HCC.^25^ Known as extracellular glycoproteins interacted with ECM substrate and cell adhesion molecules, FBLNs are considered to be critical for organ development and elastic fiber formation.^17^ Among them, FBLN2, a key pro-fibrotic factor, plays a crucial role in promoting the process of liver cirrhosis.^18^ Yet, as one of the 64 upregulated RNAs associated with solid tumor-derived metastasis,^26^ the function of FBLN2 in several tumors appears to be controversial. For example, high FBLN2 is related to adverse pathologic variables including tumor invasion and metastasis in urothelial carcinoma.^8^ Fibulin-2 is also a driver of migration and invasion of lung adenocarcinoma cells.^9^ However, FBLN2 impairs the invasion and migration in breast cancer.^16^ Considering the pathological progression from cirrhosis to HCC, the role of FBLN2 in HCC aroused our interest. There is a paucity of study evaluating the role of FBLN2 in HCC. Literature has reported that an upregulated FBLN2 is discovered in HCC samples, which is validated in our research.^21^ Depending on the date of HccDB database and HPRD database, previous studies hypothesized FBLN2 is related with poorer prognosis of HCC patients^19^ and may contribute to apoptosis in HCC.^20^ In view of the limited experimental research data on FBLN2 in HCC, we investigated the effect of FBLN2 in HCC and provided testable evident for previous hypotheses. In our study, data from public databases revealed upregulated expression of FBLN2 in HCC tissue compared to normal liver tissues, with is further validated by IHC of HCC tissues from HCC patients. According to epidemiologic studies, a precious history of liver cirrhosis was associated with an elevated expression of FBLN2, which corroborated previous researches.^18^ However, the groups are small with significant differences in age across them. There might be biases in the current study, thus, it is important to verify this result by expanding the sample size. And most HCC cell lines exhibited significantly higher levels of FBLN2 as compared to non-malignant hepatic cell line. Cellular biological experiments indicated that FBLN2 facilitated proliferation, cell cycle progress, and hinder cell apoptosis of HCC cells. Moreover, in vivo assessment in the xenograft mouse model manifested that knockdown of FBLN2 inhibited HCC growth. In conclusion, the roles of FBLN2 in proliferation, apoptosis, and cell cycle of HCC were examined by in vitro and in vivo xenograft experiments. Accordingly, we concluded that FBLN2 acts as an oncogene in HCC.

Although the oncogenic role of FBLN2 in the development of HCC is well established, the underlying mechanism responsible for the effect of FBLN2 needs to be further addressed. As a well-known signature pathway of cancer cell growth, the Ras-MEK-ERK1/2 pathway stimulates several transcription factors (e.g., Elk-1, c-Jun), implicated in apoptosis, cell cycle progression, proliferation, cell migration, and differentiation.^27^ In 50%-100% of primary HCC, Ras/MAPK pathway is activated and associated with poor patient prognosis.^28^ Blocking the Ras-MEK-ERK1/2 signaling pathway was proved to have multiple anticancer properties.^27^ Here in our study, the Ras-MEK-ERK1/2 signaling pathway was found to be activated in HCC cells following the overexpression of FBLN2, which is in complete accord with a series of diseases such as non-small-cell lung cancer^29^ and cardiac hypertrophy.^30^ Thus, it could be speculated that FBLN2 plays a key role in the regulation of the MAPK signaling pathway in a wide range of pathological processes, such as cell proliferation and apoptosis, which further highlights that FBLN2 could be used as a promising therapeutic target in oncology.

It should not be neglected that our study still has some unsatisfactory limitations. Previous studies demonstrated that FBLN2 was closely associated with either the promotion or inhibition of cancer invasion and migration.^5^ Previous study showed that FBLN2 protein digestion was implicated in the migration and invasion of breast cancer cells.^16^ However, in the present study, the role of FBLN2 in cell invasion and migration was found to be insignificant in HCC cells. Meanwhile, the mechanisms attributing to contrary faces of FBLN2 in cell migration and invasion warrant additional studies. In addition, we have only made a preliminary exploration of the mechanism of FBLN2 responsible for these differences and further research is needed to investigate the underlying mechanisms to gain better insights. In addition to this, the specific binding sites for these related molecules remain to be determined. On the other hand, immunohistochemistry from a larger sample size of patient clinical specimens is still needed to determine the prognostic impact of FBLN2 on HCC.

In conclusion, FBLN2 might be able to serve as a biomarker of HCC. Fibulin-2facilitated tumor cell proliferation and regulated cell apoptosis and cell cycle arrest, which was mediated via modulation of the Ras-MEK-ERK1/2 signaling pathway. Altogether, the oncogenic role of FBLN2 in HCC, established in the present study, provided a new research perspective that could be exploited for the development of novel target therapy in the future.

## Figures and Tables

**Supplementary Table 1.** Clinical Characteristics of 9 HCC Patients

Patient Characteristics	Patients (n = 9)
Gender	
Male	8
Female	1
Age (years)	
≤55	2
>55	7
AJCC stage	
1 + 2	6
3 + 4	3
Tumor size (cm)	
≤3	6
>3	3
T (tumor)	
T1/T2	7
T3/T4	2
Liver cirrhosis	
Yes	6
No	3

**Supplementary Table 2.** Correlation Between FBLN2 Expression and HCC Clinicopathologic Features

Patient Characteristics	FBLN2 Expression Levels		*P*
	High Expression (n = 5)	Low Expression (n = 4)	
Gender			
Male	5	3	.048*
Female	0	1	
Age (years)			
≤55	0	2	>.05
>55	5	2	
AJCC stage			
1 + 2	3	3	>.05
3 + 4	2	1	
Tumor size (cm)			
≤3	3	3	>.05
>3	2	1	
T (tumor)			
T1/T2	3	4	>.05
T3/T4	2	0	
Liver cirrhosis			
Yes	5	1	.048*
No	0	3	

*P < .05 were considered statistically significant.
